# Primordial germ cells: the first cell lineage or the last cells standing?

**DOI:** 10.1242/dev.113993

**Published:** 2015-08-15

**Authors:** Andrew D. Johnson, Ramiro Alberio

**Affiliations:** 1 School of Life Sciences, Queen's Medical Centre, University of Nottingham, Nottingham NG7 2UH, UK; 2 School of Biosciences, Sutton Bonington Campus, University of Nottingham, Loughborough LE12 5RD, UK

**Keywords:** Amphibian embryo, Evolvability, Germ plasm, Mammalian embryo, Pluripotency, Primordial germ cell, PGC

## Abstract

Embryos of many animal models express germ line determinants that suppress transcription and mediate early germ line commitment, which occurs before the somatic cell lineages are established. However, not all animals segregate their germ line in this manner. The ‘last cell standing’ model describes primordial germ cell (PGC) development in axolotls, in which PGCs are maintained by an extracellular signalling niche, and germ line commitment occurs after gastrulation. Here, we propose that this ‘stochastic’ mode of PGC specification is conserved in vertebrates, including non-rodent mammals. We postulate that early germ line segregation liberates genetic regulatory networks for somatic development to evolve, and that it therefore emerged repeatedly in the animal kingdom in response to natural selection.

## Abstract (precis)

**Summary:** In this Hypothesis article, Johnson and Alberio propose that the determinative mechanisms for PGC specification in most model systems evolved to promote speciation and evolvability, not to maintain the germ line.

## Introduction

Germ cells and somatic cells (or soma) engender perhaps the most basic division of cellular function in metazoan biology. Germ cells are the source of heritable genetic variation, and they produce the totipotent zygote from which embryogenesis commences. The somatic cell lineages, or soma, develop under the control of the zygotic genome and determine the fitness of genetic innovations in response to selection. In this way, the germ line:soma relationship coordinates metazoan evolution (Johnson et al., 2011). The germ line is distinguished as an independent cell lineage when primordial germ cells (PGCs) are specified during embryogenesis. However, the mechanisms that direct PGC specification are not conserved across the animal kingdom or even, in some cases, between closely related species. For example, cytoplasmic germ cell determinants, known as germ plasm, have evolved in the oocytes of many animal lineages, including frogs (Houston and King, 2000), teleost fish (Raz, 2003), worms and flies (Nakamura and Seydoux, 2008), and ascidians (Shirae-Kurabayashi et al., 2011) (Table 1). Germ plasm inhibits transcription in nascent PGCs, presumably to inhibit differentiation to a somatic fate. However, germ plasm is not conserved; it arose throughout the animal kingdom by convergent evolution (Extavour and Akam, 2003; Johnson et al., 2001, 2003b). In other species, PGCs are specified without germ plasm; notable examples of such species are sea urchins and mice. Sea urchin PGCs accumulate maternally deposited germ line transcripts and are transcriptionally inert (Swartz et al., 2014), similar to PGCs with germ plasm. However, in mouse embryos germ line restriction is induced in transcriptionally active cells by expression of the transcription factor Blimp1 (Prdm1 – Mouse Genome Informatics), which inhibits mesoderm specification in nascent PGCs through repression of a specific set of genes (Ohinata et al., 2005).

**Table 1.**
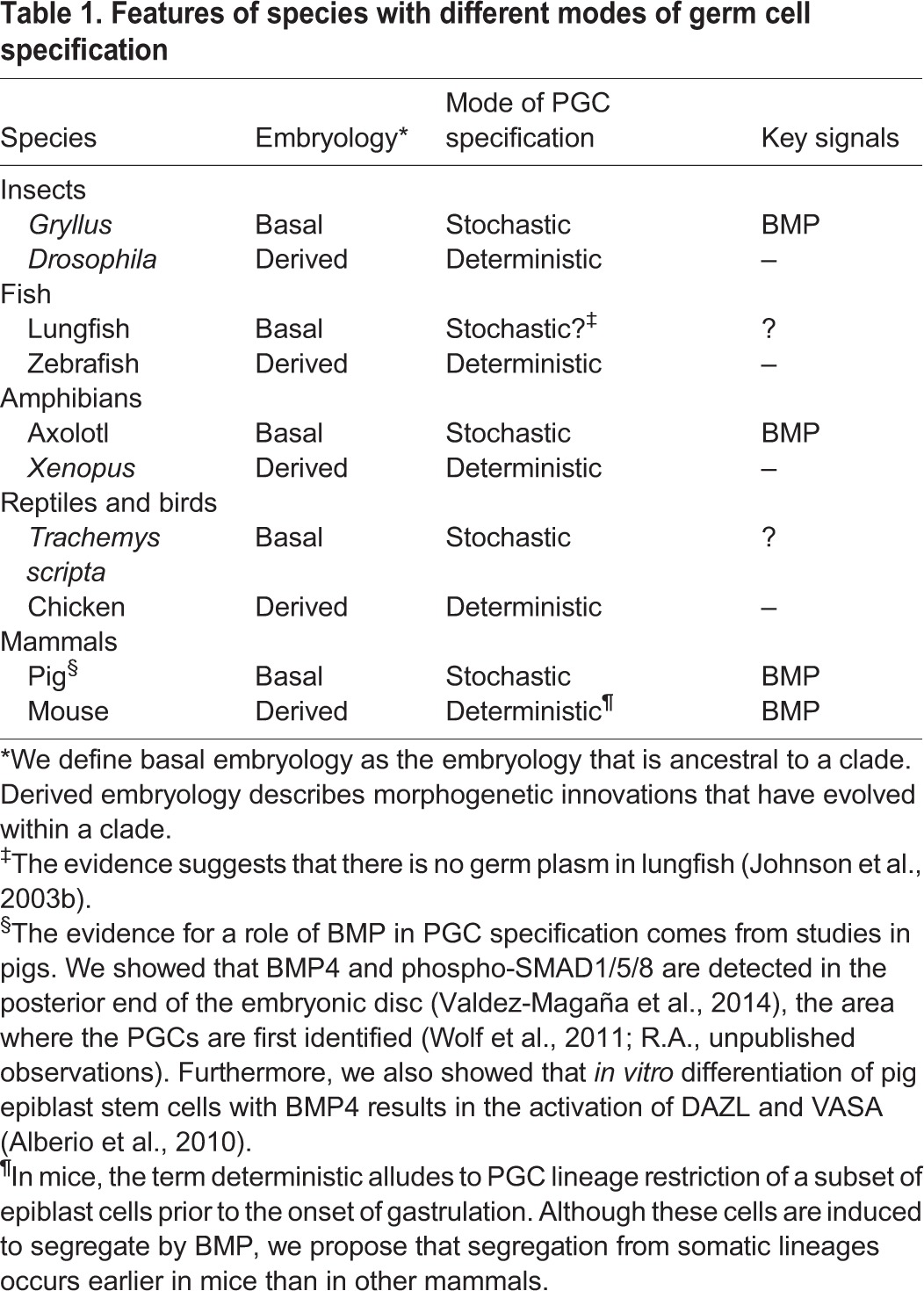
**Features of species with different modes of germ cell specification**

It is clear from this analysis that germ line determination is achieved through a diverse array of mechanisms, many of which act in the very early phases of development. Indeed, regardless of whether they are specified by factors of maternal or zygotic origin, in most commonly studied animal models PGCs are the first cells to undergo lineage restriction during embryogenesis. How or why these ‘deterministic mechanisms’ evolved is unknown, but a long-standing consensus is that they are required to safeguard the unique properties of germ cells (Blackler, 1970; Seydoux and Braun, 2006). Whether this is the case, or why it would be necessary, endures as a fundamental problem in biology.

In this Hypothesis, we discuss the timing and mechanisms of PGC specification in the vertebrate lineage. Several distinct modes of PGC specification are known to exist in vertebrates (Johnson et al., 2003b, 2011), yet vertebrate natural history is unambiguous. This enables the evolutionary history of individual mechanisms for PGC specification to be deduced within an accurate phylogenetic context. We propose that late specification, as described for axolotls in the ‘last cell standing’ model (Chatfield et al., 2014), represents a conserved paradigm for vertebrate germ line development. However, we also propose that early-acting deterministic mechanisms evolved repeatedly to accelerate germ line restriction, not to protect inherent properties of the germ line. We further speculate that these mechanisms evolved because precocious segregation of the germ line from the soma promotes the evolution of embryological innovations that enhance speciation and lead to accelerated development, both of which are favoured by selection. We discuss this hypothesis within the context of the fundamental relationship between the germ line and soma.

## Established models of PGC specification

**Fig. 1.**
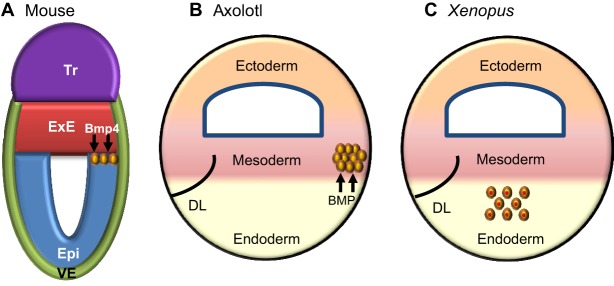
**Primordial germ cell specification in mice, axolotls and frogs.** (A,B) In mice (A) and axolotls (B), BMP is a key inductive molecule required for the specification of germ cell precursors (Lawson et al., 1999; Chatfield et al., 2014). (C) In *Xenopus*, germ plasm components inherited from the egg are passed to a group of cells in the endoderm of the developing embryo that become PGCs (Blackler, 1962). DL, dorsal lip; Epi, epiblast; ExE, extra-embryonic ectoderm; Tr, trophectoderm; VE, visceral endoderm. Yellow circles depict primordial germ cell precursors. In the case of *Xenopus*, the red centre depicts the germ plasm.

Among vertebrate developmental models, the mechanisms that direct PGC commitment in *Xenopus* and mice are understood at the finest levels of detail. In *Xenopus*, PGCs are derived from blastomeres that inherit germ plasm, an aggregate of germ cell determinants localized in the vegetal cortex of the egg (Houston and King, 2000). Germ plasm inhibits transcription and translation in nascent PGCs (Venkatarama et al., 2010; Lai et al., 2012), and this prevents germ cell precursors from responding to somatic specification signals. Importantly, because germ plasm is inherited from the egg, PGCs comprise the first cell lineage to be established during *Xenopus* embryogenesis: PGC specification occurs prior to specification of the somatic cell lineages (Fig. 1C).

In mammals, early embryogenesis is more complex than in amphibians in that it includes the development of extra-embryonic tissues. The first cell fate decision in mammalian development distinguishes the trophoblast lineage, which contributes to the placenta, from the developing epiblast, which will give rise to both the germ line and somatic cells of the embryo (Rayon et al., 2014). In mouse embryos, derivatives of the trophoblast lineage play a focal role in PGC specification (Fig. 1A). For instance, bone morphogenetic protein 4 (Bmp4) signals emanating from the extra-embryonic ectoderm (ExE), a trophoblast derivative, initiate PGC specification in a small group of cells located in the proximal epiblast at around embryonic day (E) 6.25, about a day prior to the onset of gastrulation (Lawson et al., 1999). A key event triggered by Bmp4 is expression of the transcription factor Blimp1, which evokes irreversible restriction to the germ cell lineage. Germ line restriction occurs, in part, because Blimp1 represses expression of a number of genes involved in somatic development (Ohinata et al., 2005; Kurimoto et al., 2008). Parallels between the transcriptional repression functions of Blimp1 and germ plasm (in other models) have been noted elsewhere (Surani et al., 2004; Nakamura and Seydoux, 2008). Yet, an additional similarity is found in the relative timing of PGC specification. Because the net effect of Blimp1 expression is to direct irreversible germ line commitment prior to the specification of any of the somatic lineages, PGCs are the first embryonic cell lineage established in mouse development. We define this as ‘early PGC commitment’. Whether early PGC commitment is a general feature of mammalian development is, however, unclear.

For example, the embryos of non-rodent mammals do not form a structure equivalent to the ExE, and therefore the origin and timing of the signals that initiate PGC commitment are largely unknown (Saitou and Yamaji, 2010; Irie et al., 2014). In addition, recent studies suggest that cell-intrinsic events governing the specification of human PGCs are also different from those in mice, at least based on the results of *in vitro* assays. Significantly, in mice Prdm14 activation in nascent PGCs re-establishes expression of pluripotency genes, including Sox2 and Nanog (Yamaji et al., 2008). By contrast, PGC-like cells derived from human embryonic stem cells maintain NANOG expression, express very low or no PRDM14, and do not express SOX2 (Irie et al., 2015; Sugawa et al., 2015). Moreover, they initiate expression of SOX17 upstream of BLIMP1 (PRDM1 – Human Gene Nomenclature Database) activation (Irie et al., 2015). Together, these results suggest that different molecular mechanisms regulate PGC development in the embryos of mouse and humans, and it is not yet clear which, if either, of these modes is more widespread among mammals. The inductive mode of specification seen in mammals is also distinct from the cell-autonomous mode of PGC specification mediated by germ plasm in frogs, illustrating the diverse mechanisms of germ cell determination operating in vertebrate embryos.

To understand how, or why, divergent mechanisms emerged to govern germ line development, it is necessary to define the basal mechanism from which these evolved. For this purpose, we have used axolotl embryos as a model. Axolotls retain basal vertebrate traits (Box 1), so their embryos provide an experimental system from which the natural history of vertebrate developmental mechanisms can be deduced. Classic studies reported that axolotl PGCs are formed by induction from pluripotent cells (Boterenbrood and Nieuwkoop, 1973; Sutasurja and Nieuwkoop, 1974; Michael, 1984), as in mammals, and we recently elaborated the mechanism underlying their specification (Chatfield et al., 2014). In axolotls, PGCs are derived from multipotent mesodermal cells, development of which is specified by the combination of fibroblast growth factor-4 (FGF-4) and bone morphogenetic protein-4 (BMP-4) signalling (Fig. 1B). Germ cell potential is maintained in these cells by signalling through the MAP kinase (MAPK) pathway, so that disruption of MAPK signalling prior to the completion of gastrulation abrogates germ line development. Indeed, irreversible germ line commitment is not completed until early tailbud stages (Chatfield et al., 2014). These results demonstrate that the germ line in early axolotl embryos is maintained by a signalling niche, and that germ line commitment occurs after gastrulation. On this basis, we have proposed that PGC specification in axolotls is governed by a ‘stochastic’ process, similar to the mechanisms that specify development of the somatic lineages. In this model, PGC development remains only a probability until relatively late in development: if the niche changes prior to lineage restriction, potential germ cells will be diverted to an alternative fate. We define this as ‘late’ PGC specification and to describe this process we proposed the ‘last cell standing’ model, which postulates that PGCs develop from the last cells in the embryo to engage in lineage commitment (Chatfield et al., 2014).

Box 1. **Axolotls model primitive vertebrates**

The fossil record demonstrates that vertebrates occupied land as early as 395 million years ago (Niedźwiedzki et al., 2010), and that the overall morphology of these primitive tetrapods, and their aquatic ancestors, resembled that of extant urodele amphibians (Ahlberg et al., 2005, 2008). This suggests that modern urodeles retain primitive embryological mechanisms, but direct evidence for this comes from comparing the embryology of axolotls and lungfish. Phylogenetic analysis demonstrates that lungfish represent the closest living relative of the tetrapod ancestor (Brinkmann et al., 2004; Amemiya et al., 2013), and the yolky embryos of lungfish superficially resemble those of urodeles (Kemp, 1981; Wourms and Kemp, 1982). More importantly, perhaps, detailed comparative analysis shows that the embryos of lungfish and axolotls share conserved morphogenetic traits that are basal to vertebrates, most notably a conserved mechanism for gastrulation (Shook and Keller, 2008). It has been proposed that these basal embryological mechanisms were conserved as amniotes evolved from urodele-like amphibians (Shook et al., 2002; Shook and Keller, 2008; Bachvarova et al., 2009b), and that this explains the extraordinary retention of the body plan of urodeles and reptiles (lizards, crocodilians, etc.) across a transition that required the evolution of amniote embryology (Johnson et al., 2011). This feature presents axolotls as a well-suited model for the amphibious ancestor to mammals. In this regard, basal amphibian embryology diverged in the anuran lineage (Shook and Keller, 2008; Johnson et al., 2003b) after they diverged from a urodele-like ancestor ∼260 million years ago (Anderson et al., 2008; Zhang and Wake, 2009). Indeed, anurans evolved a diverse range of morphogenetic patterns (Keller et al., 2000); the absence of similar variation in extant urodeles (Keller, 2000) suggests that anuran embryology is endowed with a unique capacity to withstand embryological innovations, as discussed elsewhere (Johnson et al., 2003b).

Because axolotls retain basal vertebrate traits, we propose that the last cell standing model may define the basal state for PGC specification in embryos of all vertebrate lineages, mammals notwithstanding. In mammals, as in many phyla, two modes of PGC specification apparently exist: the well-described mechanism in mouse and a distinct mechanism that appears to operate in human. We posit that a unifying logic must oversee the evolution of developmental mechanisms throughout the animal kingdom. Thus, to shed light on why different mechanisms for PGC specification may have evolved in mammals, we first consider a precedent wherein the problem of divergent PGC specification has been integrated into a model for amphibian evolution.

## The vexing problem of germ cell specification in vertebrates

The study of germ cell development in amphibians has a long and contentious history. By the turn of the twentieth century, PGCs had variably been identified in either the endoderm or mesoderm of amphibian embryos, leading to conflict concerning their origin. Humphrey (1925) quelled disagreement in the field by confirming a dual origin for amphibian PGCs, entailing their derivation from mesoderm in urodeles (salamanders) and endoderm in anurans (frogs). Nonetheless, he postulated that the mechanism governing PGC specification must be the same in both amphibian lineages, with the different origins of the cells resulting from divergent morphogenetic mechanisms. Interestingly, this early hypothesis illustrates a general problem in germ cell biology, which has repeatedly assumed that disparate mechanisms for PGC development must somehow represent subtle variants of a conserved process. However, as was first demonstrated in amphibians, it is now recognized that mechanisms directing the specification of PGCs can diverge even within a single clade.

Early studies on frog embryos identified germ plasm in several species (Blackler, 1958), indicating that it is indeed conserved in the anuran lineage. Deletion and transplantation experiments later demonstrated that germ plasm acts as a determinant of germ line development (Blackler, 1962; Smith, 1966), and recent work shows that transplanted germ plasm can induce ectopic PGCs (Tada et al., 2012). In *Xenopus*, germ plasm acts by inhibiting transcriptional elongation in PGCs, and by repressing translation of maternally inherited somatic determinants (Venkatarama et al., 2010; Lai et al., 2012). Together, these activities ensure maintenance of germ cell identity amidst intrinsic and extrinsic somatic influences. Significantly, this repressed physiological condition is imposed prior to transcriptional activation at the midblastula transition and it persists through the completion of gastrula stages (Venkatarama et al., 2010) to ensure the early lineage commitment of PGCs.

For generations the idea of a continuous germ cell lineage, development of which was predetermined in each new generation by the inheritance of maternally inherited determinants (Blackler, 1970), reigned as the textbook model for vertebrates. However, Nieuwkoop and colleagues challenged this concept based on their work with axolotl embryos. They showed that PGCs could be induced from the animal cap of axolotl embryos by mesoderm-inducing signals (Boterenbrood and Nieuwkoop, 1973; Sutasurja and Nieuwkoop, 1974). Moreover, they could not detect germ plasm in axolotl embryos (Ikenishi and Nieuwkoop, 1978). On these grounds, it was concluded that PGCs in axolotls arise from ‘unspecialized cells’, in contrast to the predetermined germ cells of frogs (Nieuwkoop and Sutasurya, 1979), and this concept fostered conflict within the field.

The possibility that divergent modes of PGC specification could exist within an individual phylum, such as amphibians, was difficult to reconcile amidst prevailing knowledge of the time. Thus, it was largely discounted. For example, it was proposed that germ plasm might assume an alternative form in urodeles that predestines a subpopulation of mesodermal cells to the germ line (Smith et al., 1983; Michael, 1984). Alternatively, Nieuwkoop and Sutasurya (1976) postulated that frogs and salamanders evolved from different lineages of fish; at this time, the phylogeny of extant amphibians was less certain. However, it is now known that extant amphibians are monophyletic (i.e. they share a common ancestor) and are derived from an ancestor with urodele-like features (Box 1). Working within this phylogenetic context, we revisited PGC development in axolotls to clarify whether or not anurans and urodeles share a common mechanism for PGC specification (Johnson et al., 2001). Like Nieuwkoop and colleagues, we were unable to detect germ plasm in axolotls, and proposed that inductive specification of PGCs is conserved in vertebrates. However, by this time germ plasm had been identified in the embryos of zebrafish (Knaut et al., 2000) and chick (Tsunekawa et al., 2000), leading us to propose that germ plasm evolved *de novo* in frogs, and other vertebrates, by convergence (Johnson et al., 2001). This hypothesis raised an intriguing question, however. Namely, what would drive the evolution of germ plasm?

## Germ plasm liberates evolution of somatic development

The evolution of a trait by convergence implies that it conveys an advantage under selection. At the species level, direct evidence for the advantage of germ plasm is clear. Animal lineages that evolved germ plasm contain between ten and 1000 times more species than sister clades that employ inductive specification (Crother et al., 2007; Johnson et al., 2011). In addition, species with a predetermined germ line evolve more rapidly (Evans et al., 2014). These findings indicate that germ plasm might enhance evolvability, which is thought to be favoured by selection (Box 2).

Box 2. **Selection for evolvability**

Kirschner and Gerhart (1998) define evolvability as “the capacity to generate heritable, selectable, phenotypic variation”. They, and others (Earl and Deem, 2004), have argued that enhanced evolvability is favoured by natural selection, although this is difficult to prove. The idea that evolvability is favoured by selection is supported by experimental evidence (Colegrave and Collins, 2008), but the concept nevertheless remains controversial because it predicts selection for a trait that acts in future generations, which does not necessarily benefit an individual in the present, i.e. it suggests a pre-adaptation. We, however, favour the hypothesis that enhanced evolvability arises within populations to promote propagation of the germ line, in accord with Dawkins' principles (Dawkins, 1976). In our view, conditions that improve the capacity of the soma to evolve as a more diverse and adaptable vehicle for germ line transmission would be favoured by selection. This might include enhancement of an individual's survival beyond reproductive maturity, but this would arise only as a secondary adaptation. Indeed, the evolutionary significance of traits that benefit the soma, from this perspective, should only be considered within the context of their ability to affect germ line propagation.

It is reasonable to assume that enhanced evolvability results from effects on somatic development, and therefore that germ plasm may enhance the potential to evolve somatic innovations. If so, because germ plasm is maternally inherited, it would be expected to exert its influence exclusively in the early stages of development. In support of this, we have concluded that the genetic innovations leading to the anteriorized morphology of frog embryos could not have evolved prior to the evolution of germ plasm (Johnson et al., 2003b), suggesting that germ plasm may have played a causal role in the evolution of anurans. Conversely, we also proposed that the stochastic mode of PGC specification, found in axolotls, inhibits the potential of somatic innovations to evolve, resulting in conservation of the molecular mechanisms governing development of the basal vertebrate body plan (Johnson et al., 2003b).

**Fig. 2.**
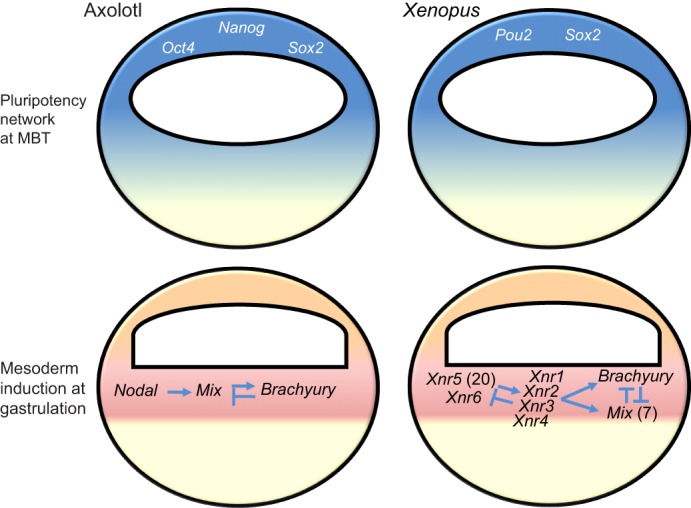
**Gene regulatory networks for early development have been modified in *Xenopus*.** Pluripotency is established at the midblastula transition (MBT) in amphibians. Frogs have lost key components of the pluripotency network (specifically, Nanog and Oct4) that are conserved between urodele amphibians and mammals. Furthermore, the GRN that regulates mesoderm specification in *Xenopus* has expanded. By comparison with the GRN for mesoderm in axolotl, the *Xenopus* GRN shows novel features, including expansion of the Nodal (25 orthologues, denoted *Xnr*) and Mix (seven orthologues) gene families, Nodal gene expression prior to the midblastula stage, and altered topological relationships. See Swiers et al. (2010) for details.

How germ cell determination might dictate the pattern of vertebrate evolution is becoming clearer through comparative analysis of axolotl and *Xenopus* development. For example, it has long been known that the animal caps of axolotl and *Xenopus* embryos harbour different developmental potential, because the animal caps of frog embryos cannot support the development of PGCs (Michael, 1984). The genetic machinery that underpins this difference is now understood. Development of cells in the animal hemisphere of axolotl embryos is controlled by a gene regulatory network (GRN) that includes orthologues of the transcription factor genes *Nanog*, *Sox2* and *Oct4* (Bachvarova et al., 2004; Dixon et al., 2010; Tapia et al., 2012), which also control the acquisition of pluripotency in mammalian embryos (Fig. 2). The *Xenopus* genome, however, does not encode orthologues of *Nanog* (Hellsten et al., 2010). *Oct4* is also not encoded (Frankenberg and Renfree, 2013), though the related *pou2* family of transcription factors is expanded in *Xenopus* and subfunctionalized activities of these factors apparently assumed control over some aspects of pluripotency (Morrison and Brickman, 2006; Livigni et al., 2013). Nonetheless, the pluripotency GRN is not conserved in *Xenopus*. The deletion of pluripotency genes in the anuran lineage must have occurred after divergence from urodeles, about 260 million years ago (Zhang and Wake, 2009). We propose that this was enabled by the evolution of germ plasm, which transitioned PGC specification from zygotic control to cell-autonomous regulation by inherited cytoplasmic factors. As a consequence, passage through a pluripotent state that can support germ cell development was rendered dispensable.

Comparative analysis also indicates that the GRN regulating mesoderm development in *Xenopus* is an innovation. The *Xenopus* mesoderm GRN contains >25 variants of *Nodal*-related genes in addition to seven copies of the homeodomain transcription factor *Mix*, which transduces signalling downstream of *Nodal* (Loose and Patient, 2004; Takahashi et al., 2006; Wardle and Smith, 2006). By comparison, the GRN for axolotl mesoderm contains single copies of *Nodal* and *Mix* (Swiers et al., 2010), which is similar to the mesoderm GRN of mammals (Fig. 2). Chatfield et al. (2014) have argued that the stochastic mechanism for PGC specification in urodeles precludes expansion of the mesoderm GRN, as configured in *Xenopus*, because it would terminate germ line development. This conjecture is based on the signalling requirements for PGC specification in axolotls. Axolotl PGCs develop in the ventral marginal zone (VMZ) adjacent to the precursors of blood cells. Nodal and fibroblast growth factor (FGF) signals compete for a common pool of precursor cells, to direct blood or PGC specification, respectively (Chatfield et al., 2014). Crucially, titration experiments showed that excess levels of Nodal can overwhelm the effects of FGF and induce blood at the expense of PGCs. These results indicate that the levels of Nodal signalling in axolotl embryos must exist in equilibrium with FGF levels. We postulate that this equilibrium acts as a constraint on the ability to evolve an expanded Nodal gene family, as observed in *Xenopus*. Likewise, overexpression of Mix, which transduces Nodal signalling, eliminates PGCs (Chatfield et al., 2014), suggesting that evolution of the Mix gene family is also under constraint. Together, these findings suggest that the evolution of germ plasm was a necessary precondition for the innovations observed in the *Xenopus* mesoderm GRN. As discussed above, germ plasm inhibits the response of nascent PGCs to external stimuli (Venkatarama et al., 2010). Therefore, anuran PGCs are no longer maintained by homeostatic signalling, so the mechanisms that govern somatic development should be free to evolve independently of a detrimental influence on development of the germ line.

In our view, liberated from constraints, selection would favour the evolution of molecular mechanisms that mediate more rapid somatic development. The ability to evolve novel regulatory circuits would also enhance speciation. The last cell standing model predicts opposite effects: lineage-restricted PGCs emerge after the onset of gastrulation (Chatfield et al., 2014) and their development is dependent on the maintenance of signals emitted from adjoining somatic cells, not cell-autonomous determinants. Therefore, the potential to reconfigure GRNs for early development would be inhibited. Evidence for this comes from the conservation of morphogenesis (Shook et al., 2002; Shook and Keller, 2008), and body plan, through the evolutionary interval between primitive tetrapods and mammals, which implies that early embryo patterning in the absence of germ plasm is invariant.

Although evidence for the selective advantages of germ plasm is apparent for anamniote vertebrates (frogs and fish), here we hypothesize that equivalent biological principles also apply to mammals. It is in this light that we postulate that early germ line restriction by *Blimp1*, ‘Blimping’, evolved in rodents and enhanced evolvability.

## PGC specification in mammals

Precursors of the germ line in mice are first detected by *Blimp1* expression in a small number of founders in the E6.25 proximal epiblast. By E7.25, a cluster of ∼40 *Blimp1*-expressing cells activate the germ line programme (Ohinata et al., 2005). As mentioned above, Blimp1 is a zygotic determinant of germ line development, expression of which mediates lineage restriction of pluripotent cells. It is activated in response to Bmp4 secreted by neighbouring ExE cells and it acts by repressing expression of genes that promote somatic differentiation (Kurimoto et al., 2008). Following lineage restriction, PGC precursors initiate germ cell specification by activating Prdm14 and AP2γ (Tfap2c – Mouse Genome Informatics). Prdm14 is the first germ cell gene to be expressed and plays crucial roles in two successive events characterizing the germ cell programme: reacquisition of pluripotent potential and epigenetic reprogramming (Weber et al., 2010; Yamaji et al., 2008). Indeed, when these factors are co-expressed they can induce PGC-like cells in the absence of cytokines, suggesting that the tripartite network of Blimp1, Prdm14 and AP2γ is sufficient for mouse PGC specification (Magnúsdóttir et al., 2013; Nakaki et al., 2013). Prdm14 and AP2γ work in conjunction with one another to activate terminal markers of PGCs, such as Nanos3 and Dnd1, but importantly, they also cooperate with Blimp1 to repress somatic genes and some epigenetic modifiers. Indeed, although expression of the mRNA encoding *T*, a mesodermal marker, is detected in mouse PGC precursors, T protein is first detected in E6.5 early primitive streak cells (Kispert et al., 1994; Inman and Downs, 2006). Thus, we would argue that *T* expression at the time when Blimp1 is detected in posterior proximal epiblast cells (E6.25) reflects the identity of a cell population that is about to initiate a mesodermal programme in this region of the primitive streak, suggesting that Blimp1 prevents their commitment to this lineage.

During germ cell specification in mice, expression of pluripotency markers is restored following the reactivation of Nanog and Sox2 in Oct4 (Pou5f1)-expressing PGC precursors. The expression of these three factors is essential for PGC development (Kehler et al., 2004; Chambers et al., 2007; Campolo et al., 2013). Although the specific roles of these factors during germ cell development are unclear, it is thought that their expression confers latent pluripotency to the germ line. This has been most clearly demonstrated by the derivation of pluripotent embryonic germ cells (EGCs) from PGCs (Matsui et al., 1992; Labosky et al., 1994; Leitch et al., 2010). The parallels between naïve embryonic stem cells (ESCs) and PGCs have led to the suggestion that these cell types are linked in their molecular regulation. In the case of PGCs, this regulatory network is thought to protect them from somatic-inducing signals during the extensive epigenetic reprogramming they undergo (Leitch and Smith, 2013).

**Fig. 3.**
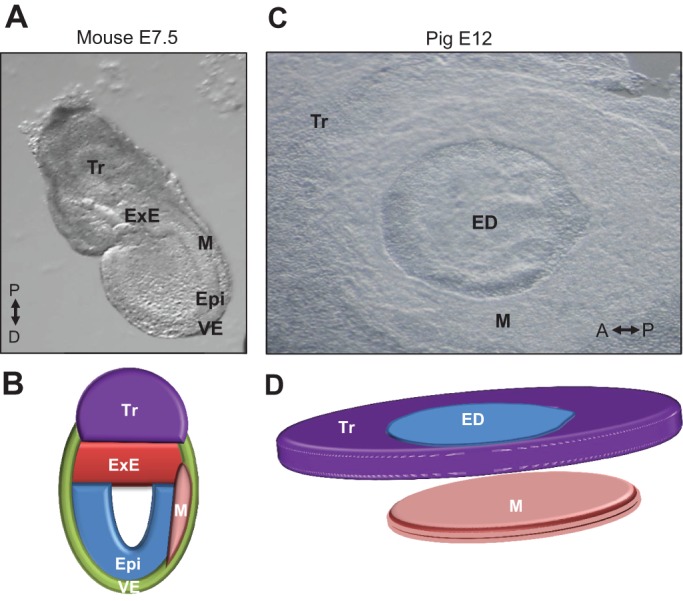
**Differences in early embryo morphology within mammals.** (A-D) In mouse (A,B), the egg cylinder forms as a result of the cavitation of the embryo after the formation of the extra-embryonic ectoderm. In most other mammals, the epiblast does not undergo cavitation and forms a flat embryonic disc that is surrounded by extra-embryonic membranes (C,D). An image of a pig embryo (C) around the onset of gastrulation shows the configuration of a typical non-rodent mammalian embryonic disc surrounded by trophectoderm (or trophoblast). ED, embryonic disc; Epi, epiblast; ExE, extra-embryonic ectoderm; M, mesoderm; Tr, trophoblast; VE, visceral endoderm. The proximal (P)-distal (D) and anterior (A)-posterior (P) axes are indicated.

Whether the role played by Blimp1 in restriction of epiblast cells to the germ line is conserved in other mammals has not been fully elucidated. However, the absence of ExE in non-rodent embryos (human, pig, rabbit, etc.), the primary source of the inducer Bmp4 in mice, suggests that a different mechanism for germ cell specification must be at play (Fig. 3). In fact, in other mammals (such as rabbit and pig) Bmp4 is not detected prior to the onset of mesoderm formation (Hopf et al., 2011; Valdez Magaña et al., 2014). These observations suggest that early germ line restriction is an innovation of rodents that develop extra-embryonic tissues (such as ExE) producing germ line-inducing signals.

**Fig. 4.**
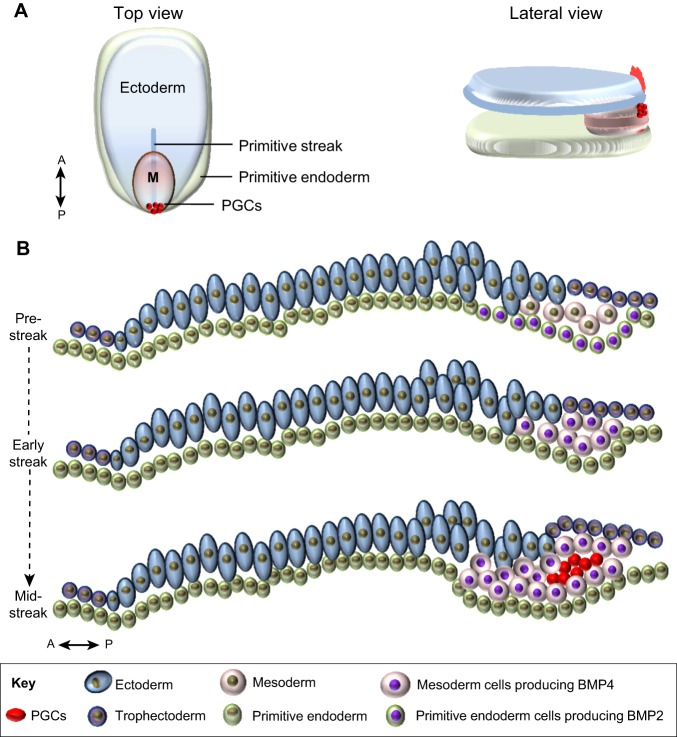
**A model for PGC specification in non-rodent mammals.** (A) Top and lateral views of the three germ layers of a peri-gastrulation embryo and the location of presumptive PGC precursors (red dots). (B) Detailed sagittal view of three developmental stages of an early to mid-gastrulation embryo. At the pre-streak stage, BMP2 produced in the posterior primitive endoderm contributes to the induction of the initial delamination of epiblast cells into the mesodermal layer. These cells expand and they start to produce BMP4 (early streak stage), contributing to the further expansion of the mesoderm. Later, at the mid-streak stage, a cluster of cells located in the midline of the embryo, posterior to the primitive streak, is induced by BMPs to initiate the germ cell programme. M, mesoderm. The anterior (A)-posterior (P) axes are indicated.

A recent report (Irie et al., 2015) showed that *BLIMP1* is activated in human PGC-like (hPGCL) cells after specification by *SOX17*, and it is suggested that its role is to inhibit the potential for somatic differentiation. Furthermore, hPGCL cells arise from precursors expressing high levels of *T* and low levels of *SOX2*, resembling posterior primitive streak-derived progenitors (Irie et al., 2015). These findings suggest that human germ cell precursors may arise from a population of posterior primitive streak-derived (i.e. post onset of gastrulation) cells that activate *BLIMP1* in response to paracrine signals. The precise combination of signals that promote germ line segregation in humans is currently unknown, but we propose that gradients of FGF, activin, BMP and Wnt signals constitute key components of a permissive (FGF/activin, Wnt) and instructive (BMP) embryological niche that ensures PGC specification (Fig. 4). Below we explain the rationale for this proposition.

### FGF and Activin A maintain expression of NANOG in germ line precursors

In human ESCs (hESCs), FGF and activin A are needed for NANOG regulation (Xu et al., 2008; Vallier et al., 2009; Yu et al., 2011). Furthermore, in pig epiblasts, in which NANOG expression is also dependent on activin A signalling (Alberio et al., 2010), NANOG is maintained in a subset of posterior epiblast cells presumed to become PGCs (Wolf et al., 2011). Importantly, these cells are located at the midline of the embryo (Wolf et al., 2011), apparently delaminating from posterior streak cells, highlighting this region as a potential niche for germ cell specification. These data are therefore consistent with a model whereby FGF and activin A act to regulate NANOG expression in the future PGCs of non-rodent mammals.

### WNT signalling is essential in maintaining germ line competence in posterior streak cells

Recent evidence in mice shows that Wnt3 activates *T*, which in turn contributes to the sharp upregulation of *Blimp1* in the PGC cluster of E7.0 embryos (Aramaki et al., 2013), indicating that Wnt3 contributes to maintaining a germ line competent population in the posterior primitive streak. Importantly, hESCs expressing high levels of endogenous WNT acquire primitive streak characteristics and have increased propensity for differentiation into mesendodermal cells (Blauwkamp et al., 2012). Moreover, hPGCL cells can be efficiently generated from hESCs maintained in medium containing the GSK3β inhibitor CHIR990021 (Irie et al., 2015). Based on these observations, we suggest that WNT signalling is a key component of the PGC niche.

### BMP signalling triggers the germ cell programme from mesodermal progenitors

Previous studies have shown that BMP4 can induce germ cell markers in human and pig stem cells (Kee et al., 2006; Alberio et al., 2010), and BMP2 and BMP4 efficiently induce hPGCL cells from hESCs (Irie et al., 2015). The role of BMPs as inductive signals in embryonic disc (ED)-forming species is consistent with evidence from studies in rabbit and pig embryos showing that BMPs are highly expressed in the primitive endoderm as well as in early mesodermal cells during gastrulation (Hopf et al., 2011; Valdez Magaña et al., 2014). This evidence suggests that posterior primitive streak cells, which delaminate from the ED in response to mesoderm-inducing signals from the primitive endoderm, such as BMP2 (Hopf et al., 2011; Valdez Magaña et al., 2014), may contribute to a niche of precursors expressing BMP4 (Fig. 4). We propose that within this niche of BMP2/4-expressing cells, a subset of mesoderm progenitors activates early PGC markers. As the niche expands, new cells are recruited to the germ line, and others segregate towards a mesoendodermal fate. The combinatorial actions of these cytokines induce a PGC niche to which cells are recruited from a pool of multipotent progenitors.

### Key differences between the mouse and other mammalian PGC programmes

This model highlights a number of key features of PGC specification in non-rodent mammals. First, Nanog expression seems to be retained in precursors with germ line potential; second, Blimp1 is first activated in specified PGC precursors, rather than in epiblast cells; and third, Sox2 expression, which is maintained in mouse migratory and gonadal PGCs (Campolo et al., 2013), is extinguished from specified non-rodent PGCs and is not restored at later stages (de Jong et al., 2008; Perrett et al., 2008). This hypothetical molecular context would contrast markedly with the observations in mice.

## The evolution of rodents

A comparison with the emerging mechanism for PGC specification in other mammals suggests that in mouse embryos the repressive functions of Blimp1 may have been co-opted to trigger precocious lineage restriction of PGC precursors. It has been proposed that the net effects of Blimping resemble those of germ plasm (Surani et al., 2004; Nakamura and Seydoux, 2008), raising the possibility that these effects evolved convergently. Interestingly, early germ line determination may have been enabled by the evolution of a novel foetal membrane, the ExE, which expresses high levels of Bmp4. The premature expression of Bmp4 has been suggested to facilitate cavitation of the egg cylinder, which is also unique to rodents, by promoting apoptosis (Coucouvanis and Martin, 1999). The association of embryological innovations with Blimping suggests that mouse embryos may have evolved in response to the same selective pressures as the embryos of anamniote species that contain germ plasm. The likelihood of this is supported further by a comparison between embryogenesis, and the reproductive strategies, of mice with those of basal rodents.

**Fig. 5.**
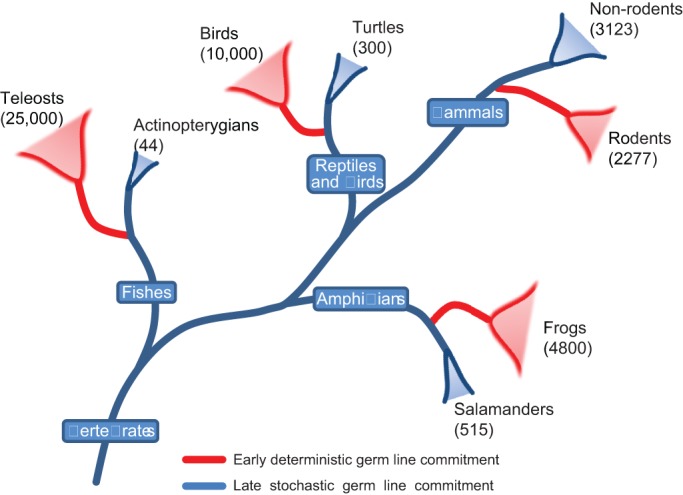
**A correlation between early germ line commitment and enhanced speciation.** All frogs studied contain germ plasm (Nieuwkoop and Sutasurya, 1979). All teleost embryos are likely to contain germ plasm (Herpin et al., 2007; Knaut et al., 2002). Also, all turtle species that have been studied use the inductive mode (Bachvarova et al., 2009a,b). The situation for other reptiles is more ambiguous. Among mammals, the rodent lineage accounts for ∼40% of all species. Numbers in brackets indicate the number of species.

*Lagostomus maximus* (plains vizcacha) is a basal South American rodent. It is a large animal (5-8 kg) with a mean gestation period of 153 days and litters of only two (Weir, 1971). These animals develop much slower than mice, and with longer generation intervals, leading to reduced prolificacy. Furthermore, the plains vizcacha embryo develops a flat ED rather than an egg cylinder (N. Leopardo and A. Vitullo, personal communication) (for comparison, see Fig. 3), indicating that the egg cylinder evolved after the rodent lineage diverged from other mammals. Following the same logic regarding evolvability, as described above for amphibians and other vertebrates, it is possible to draw correlations when analysing the number of extant mammalian species. Of the ∼5400 species of mammals, 2277 species (∼40%) are rodents (Fig. 5). More surprisingly, ∼61% of these belong to the superfamily Muridae (mice, rats and hamsters). Species within this superfamily belong to the most recently evolved rodents (Fabre et al., 2012), and they show embryological and reproductive features that are unique to this order, such as short generation intervals (∼21 days), large litters and small body size. Finally, rodents are evolving more rapidly than other species of mammals (Adkins et al., 2001). Whether Blimping of the presumptive germ cells occurs in species other than mice is uncertain. Nevertheless, based on the evident enhancement of evolvability within Muridae, we speculate that Blimping evolved in response to the same selective pressures that drove the evolution of germ plasm in anamniotes, and that early segregation of the germ line evolved repeatedly in metazoans as a common response to selective pressures encountered throughout the animal kingdom.

## “A hen is an egg's way of making another egg” (Butler, 1878)

Anne McLaren wrote: “We are taught in school that the function of the germ cells is to reproduce the species; in other words that an egg is a chicken's way of making another chicken. Samuel Butler turned this thought upside down and asserted that a chicken was an egg's way of making another egg” (McLaren, 1980). In this work she did not endorse one concept or the other. But Butler's view is biologically accurate. Indeed, the genotype of each new generation is unique, not conserved, and this is the driving force of speciation. Thus, put another way, the soma evolved to perpetuate the germ line, not the other way around*.* This fundamental biological principle (Dawkins, 1976) should underpin any explanation for how, or why, developmental mechanisms evolved. When the germ line:soma relationship is viewed this way, somatic traits evolve under selection to propagate the germ line more efficiently, not to enhance the survival of a species.

Nieuwkoop and Sutasurya divided the animal kingdom into two categories depending on whether germ cells appear early or late in embryogenesis (Nieuwkoop and Satasurya, 1981). However, their survey preceded the elucidation of PGC determination in mice, and so they assumed that PGCs are formed late in mammalian development. However, current data suggests that early germ line restriction has evolved in mammals as well, and therefore precocious germ line commitment by determinants has evolved independently in every order of vertebrates. This suggests that early germ line commitment may be an egg's way of making a more efficient hen.

We have discussed above and elsewhere how segregating the germ line before specifying somatic germ layers might facilitate evolution in anamniotes. In mammals, we propose that precocious commitment of cells to the germ line by Blimping facilitated evolution of the embryological innovations that are found in mice and other rodents, which led to enhanced speciation and the accelerated embryogenesis that is characteristic of mice. By contrast, stochastic specification, implied by the last cell standing model, constrains the evolution of novelty in the early embryo because germ line development in this context requires a signalling niche provided by neighbouring somatic cells; perturbations to homeostatic signalling within this niche would divert PGCs to a somatic fate, terminating the germ line. Existing evidence strongly suggests that the signalling niche described above for mammalian PGCs is conserved in axolotl development (Bachvarova et al., 2001; Chatfield et al., 2014; Johnson et al., 2003a), suggesting it is conserved within the evolutionary trajectory leading to higher order phyla. Speciation within this trajectory occurs more slowly, but it includes the emergence of more complex forms of embryogenesis, for example those of reptiles and mammals. Germ plasm evolved independently in teleost fish, anuran amphibians, and in the amniote lineage leading to birds. The emergence of germ plasm is associated with the divergence of animal lineages towards new paths that evolve rapidly but do not lead to higher order phyla, suggesting that germ plasm promotes the evolution of species-rich evolutionary cul-de-sacs. We speculate that the evolution of Blimping in rodents had a similar effect (Fig. 5).

Weismann first postulated that the precursors of the germ line are fundamentally different from the cells that establish the somatic cell lineages, and this view was reinforced by the subsequent discovery of germ plasm in model organisms ranging from protostomes to vertebrates (Weismann, 1898). Historically, it has been assumed that the biological function of germ plasm was to maintain the unique properties of the germ line (Blackler, 1970; Seydoux and Braun, 2006). More recently, parallels have been drawn between germ cells and pluripotent stem cells to explain how totipotency is maintained in the germ line (Juliano et al., 2010; Leitch and Smith, 2013; Solana, 2013). However, it is now clear that even differentiated somatic nuclei can be reprogrammed to a totipotent state (Sabour and Schöler, 2012), demonstrating unequivocally that the genome of somatic cells is equivalent to that of germ cells, at least in vertebrates. Therefore, we propose a novel role for germ plasm and early germ line segregation in general.

We propose that deterministic mechanisms have evolved throughout the animal kingdom to mediate irreversible germ line commitment early in development, thus disengaging the germ line from the mechanisms that control somatic development at the outset of embryogenesis. With germ line development ensured, pre-existing genetic mechanisms could be reconfigured to promote more expeditious somatic fate decisions in the early embryo. Accelerated embryogenesis would then be favoured under selection, and this would promote rapid evolution and enhanced evolvability. Thus, we postulate that the evolution of deterministic mechanisms for early germ line commitment liberated the potential for somatic innovation; they did not evolve to protect properties inherent to the germ line.

Rapid development is a desirable experimental trait, and as a consequence early germ line commitment occurs in many experimental models. We believe that the selection of rapid development as a favourable criterion for an experimental model has therefore skewed our concept of the fundamental germ line:soma relationship. The last cell standing model proposes that germ cell potential is maintained through early development in response to signalling, not by determinants. In our view, this stochastic model, in which the precursors of germ cells can readily be converted to somatic cells, is compatible with a hypothesis in which a common population of cells was the source from which PGCs and the somatic lineages of metazoans evolved.
